# Targeted Mass Spectrometry Enables Multiplexed Quantification of Immunomodulatory Proteins in Clinical Biospecimens

**DOI:** 10.3389/fimmu.2021.765898

**Published:** 2021-11-11

**Authors:** Jeffrey R. Whiteaker, Rachel A. Lundeen, Lei Zhao, Regine M. Schoenherr, Aura Burian, Dongqing Huang, Ulianna Voytovich, Tao Wang, Jacob J. Kennedy, Richard G. Ivey, Chenwei Lin, Oscar D. Murillo, Travis D. Lorentzen, Mathangi Thiagarajan, Simona Colantonio, Tessa W. Caceres, Rhonda R. Roberts, Joseph G. Knotts, Joshua J. Reading, Jan A. Kaczmarczyk, Christopher W. Richardson, Sandra S. Garcia-Buntley, William Bocik, Stephen M. Hewitt, Karen E. Murray, Nhan Do, Mary Brophy, Stephen W. Wilz, Hongbo Yu, Samuel Ajjarapu, Emily Boja, Tara Hiltke, Henry Rodriguez, Amanda G. Paulovich

**Affiliations:** ^1^ Clinical Research Division, Fred Hutchinson Cancer Research Center, Seattle, WA, United States; ^2^ Frederick National Laboratory for Cancer Research, Frederick, MD, United States; ^3^ Cancer Research Technology Program, Antibody Characterization Lab, Frederick National Laboratory for Cancer Research, Frederick, MD, United States; ^4^ Experimental Pathology Laboratory, Laboratory of Pathology, Center for Cancer Research, National Cancer Institute, National Institute of Health, Bethesda, MD, United States; ^5^ Veteran’s Administration (VA) Cooperative Studies Program, Veteran’s Administration (VA) Boston Healthcare System (151MAV), Jamaica Plain, MA, United States; ^6^ Department of Medicine, Boston University School of Medicine, Boston, MA, United States; ^7^ Pathology and Laboratory Medicine Service, Program, Veteran’s Administration (VA) Boston Healthcare System, Jamaica Plain, MA, United States; ^8^ Department of Pathology, Harvard Medical School, Boston, MA, United States; ^9^ Department of Medicine, Dana-Farber Cancer Institute, Boston, MA, United States; ^10^ Office of Cancer Clinical Proteomics Research, National Cancer Institute, Bethesda, MD, United States

**Keywords:** immunotherapy, cancer, correlative biomarkers, mass spectrometry, immuno-MRM

## Abstract

Immunotherapies are revolutionizing cancer care, producing durable responses and potentially cures in a subset of patients. However, response rates are low for most tumors, grade 3/4 toxicities are not uncommon, and our current understanding of tumor immunobiology is incomplete. While hundreds of immunomodulatory proteins in the tumor microenvironment shape the anti-tumor response, few of them can be reliably quantified. To address this need, we developed a multiplex panel of targeted proteomic assays targeting 52 peptides representing 46 proteins using peptide immunoaffinity enrichment coupled to multiple reaction monitoring-mass spectrometry. We validated the assays in tissue and plasma matrices, where performance figures of merit showed over 3 orders of dynamic range and median inter-day CVs of 5.2% (tissue) and 21% (plasma). A feasibility study in clinical biospecimens showed detection of 48/52 peptides in frozen tissue and 38/52 peptides in plasma. The assays are publicly available as a resource for the research community.

## 1 Introduction

Immunotherapies, such as immune checkpoint inhibitors (1), therapeutic cancer vaccines (2), and CAR-T cell treatments (3–5), are revolutionizing cancer care. Substantial response rates are seen in subsets of patients with particularly responsive tumors (e.g., melanomas, hematologic malignancies), including durable responses and perhaps cures in some patients with advanced disease. However, for most solid tumors, response rates remain <15%, and we do not have sufficient predictive biomarkers to identify patients whose tumors are likely to respond (6). Additionally, a significant number of patients receiving immunotherapy experience immune-related adverse events (7) (irAEs). Many irAEs are manageable with systemic immunosuppression, but some can be life-threatening (5, 8) (e.g., encephalitis, fulminant myocarditis) or lead to treatment discontinuation. Thus, there is an urgent need to understand mechanisms of response and resistance to immunotherapies to design more efficacious and less toxic immunotherapies, to identify biomarkers to select patients for single agent vs. combination immunotherapies, and to develop biomarkers to predict and monitor irAEs.

Hundreds of immunomodulatory proteins in the tumor microenvironment sculpt the T cell response to cancer as part of the “cancer-immunity cycle,” (9) and it is critical that we be able to quantify these proteins in clinical and translational research settings to design and deliver improved immunotherapies. Protein expression is typically quantified by immunoassay methods [e.g., immunohistochemistry (10), MIBI-TOF (11), flow cytometry (12)] that depend upon antibodies that are often not monospecific (13). As a result, assay interferences are commonly encountered in complex biospecimens, compromising assay specificity, and limiting multiplexing of protein assays.

To circumvent the immunoassay limitations, analysis of RNA transcripts has been used as a surrogate for protein measurements (14); however, mRNA expression levels are not reliable predictors of the expression level of most proteins (15–18), nor do they correlate with protein activity (e.g., post-translational modifications). Furthermore, many technologies do not quantify soluble proteins in the tumor microenvironment, which can impact tumor immunity (19). Thus, we need new protein quantification technologies that complement immunoassays for quantifying human proteins to enable new scientific discoveries and medical insights.

Liquid chromatography-multiple reaction monitoring mass spectrometry (LC-MRM-MS) is an emerging protein quantification method (20) in which peptides released *via* proteolysis are quantified as stoichiometric surrogates for proteins (21, 22). In contrast to untargeted “shotgun” MS profiling-based proteomics, targeted proteomics focuses the full analytic capacity of the mass spectrometer on pre-selected peptides (and the proteins they represent) of interest. Coupling an immunoaffinity enrichment step with MRM produces immuno-MRM assays that can precisely quantify low abundance proteins (23, 24) and posttranslational modifications (25, 26). Furthermore, because the mass spectrometer is used as the detector, interferences can be readily identified and usually avoided. As a result, MRM-based assays are readily multiplexed (27, 28), and the antibodies developed for immuno-MRM need not be monospecific.

Through the incorporation of stable isotope labeled internal standards, MRM assays can be harmonized across laboratories (29, 30), even on an international stage (31). Immuno-MRM assays have been applied to make clinically relevant measurements of proteins in human cancer tissues and fluids (32), including quantifying thyroglobulin in plasma where conventional immunoassays suffer interferences (33), quantification of cardiovascular health markers in plasma (34, 35), identifying novel pharmacodynamic biomarkers (36), multiplexing quantification of inborn errors of metabolism in dried blood spots (37–39), and quantifying HER2 in tissue and bone biopsies from breast cancer patients (40–43).

In this report, we present the development and characterization of a multiplexed panel (“IO-1 panel”) of immuno-MRM assays designed to quantify immunomodulatory proteins in human tissue biopsies and biofluids. The assays target 52 peptides (46 proteins) and are part of a larger effort (44) under the Beau Biden National Cancer Moonshot (45) to accelerate scientific discovery in cancer, foster greater collaboration, and improve the sharing of data.

Fit-for-purpose bioanalytical validation was conducted for the IO-1 assay panel in tumor tissue and plasma matrices to determine performance figures of merit. The performance of the assay panel was subsequently characterized in 135 tissue biospecimens (collected from 12 different tumor types) and 45 plasma biospecimens from cancer patients. The assay panel showed robust analytical performance and the targeted peptides were widely detected in the biospecimens. Additionally, the monoclonal antibodies generated in this project were tested for use in Western blotting and protein array, and all characterization data and antibody reagents are publicly available as resources for the research community through the National Cancer Institute’s Clinical Proteomic Tumor Analysis Consortium (CPTAC) Assay Portal (46, 47) (assays.cancer.gov) and Antibody Portal (antibodies.cancer.gov).

## 2 Methods

### 2.1 Materials and Reagents

Urea (#U0631), Trizma base (#T2694), citric acid (#C0706), dimethyl sulfoxide (DMSO, #D2438), EDTA (#E7889), EGTA (#E0396), and iodoacetamide (IAM, #A3221) were obtained from Sigma (St. Louis, MO). Acetonitrile (MeCN, #A955), water (#W6, LCMS Optima^®^ grade), trifluoroacetic acid (TFA, LC-MS grade, #85183), tris(2-carboxyethyl)phosphine (TCEP, #77720), phosphate buffered saline (PBS, #BP-399-20), ammonium bicarbonate (A643-500), xylene (#422685000), and (3-[(3-cholamidopropyl) dimethylammonio]-1-propanesulfonate) (CHAPS, #28300) detergent were obtained from Thermo Fisher Scientific (Waltham, MA). Rapigest (#186001861) was obtained from Waters (Milford, MA). Formic acid (#1.11670.1000) was obtained from EMD Millipore (Billerica, MA). Lys-C (Wako, #129-02541) and sequencing grade trypsin (#V5111, Promega, Madison, WI) were used for digestion of samples. Rabbit monoclonal antibodies were produced with Epitomics/Abcam (Cambridge, MA) and Excel Biopharm (Burlingame, CA). Mouse monoclonal antibodies were produced with Precision Antibody (Columbia, MD) and the Antibody Development Facility at the Fred Hutchinson Cancer Research Center (Seattle, WA). Light (unlabeled) synthetic peptides were obtained from Vivitide (Gardner, MA) as crude (flash purified) grade. Cleavable stable isotope-labeled (heavy) peptides from Vivitide corresponding to the tryptic analyte sequence were purified >95% by HPLC, labeled with [^13^C and ^15^N] at the tryptic C-terminal Arg or Lys, and quantified by amino acid analysis (AAA). Aliquots of peptide standards were stored in 5% acetonitrile/0.1% formic acid at −80°C until use.

### 2.2 Cell Lines, Culture Conditions, and Cell Lysis

HeLa (American Type Culture Collection (ATCC), #CCL-2), Jurkat (ATCC, #TIB-152), A549 (ATCC, #CCL-185), MCF7 (ATCC, #HTB-22), and NCI-H226 (ATCC, # CRL-5826) cell lines were cultured and harvested according to manufacturers’ specifications. Briefly, cells were sub-cultured and after trypsinization, were centrifuged at 1500 rpm for 6 minutes and supernatant was removed and discarded. Cells were washed once with 1X PBS, centrifuged as before, resuspended in 1X PBS, and counted. Cells were subdivided according to desired cell number in 15 mL centrifuge tubes and centrifuged as before. Cell pellets were frozen and stored at -80°C until whole cell lysis. Cell pellets were lysed using RIPA lysis and extraction buffer (Thermo Fisher Scientific, # 89900) following the manufacturer’s protocol. Mammalian Protease Inhibitor (VWR, #VWRVM250) was added according to manufacturer’s instructions. Lysate protein concentration was estimated with a BCA assay (Pierce, #23225) as described by the manufacturer’s instructions.

### 2.3 Human Samples

FFPE tissue and plasma samples used for fit-for-purpose validation studies were commercially acquired from BioIVT (Westbury, NY). Frozen tissue and plasma samples for determination of detectability were supplied by the Clinical Proteomics Tumor Analysis Consortium (CPTAC) as anonymized samples from consenting donors collected under IRB-approved protocols. FFPE tissue was supplied by the Veteran’s Administration from consenting donors collected under IRB-approved protocols. Tissue sub-compartment cellularity (e.g. tumor, stroma, adipocytes, lymphocytes) was calculated using the HALO Tissue Classifier (Indica Labs, NM) using a machine learning algorithm that was trained to apportion the total area of a section into the following components: epithelium, stroma, adipocytes, lymphocytes, and red blood cells; the training was based on results from two pathologists using blinded stained slides from breast cancer specimens (40).

### 2.4 Protein Extraction From Frozen Tissue and FFPE Samples

To produce lysates for immuno-MRM analysis, frozen tissue was cryopulverized in a cryoPREP CP-02 (Covaris, Woburn, MA) and stored frozen until analysis. 5 μL of lysis buffer (25 mM Tris, 6 M Urea, 1 mM EDTA, 1 mM EGTA, 1% (v/v) Sigma protease inhibitor (#P8340), 1% (v/v) Sigma phosphatase inhibitor cocktail 2 (#P5726), 1% (v/v) Sigma phosphatase inhibitor cocktail 3 (#P0044)) was added for each mg wet tissue weight (up to 1000 μL). The sample was vortexed for 10-15 sec and sonicated three times in a cup horn probe (filled with ice water) at 50% power for 30 seconds. The samples were stored in liquid nitrogen until the day of digestion.

FFPE samples were processed as described previously (48). Briefly, slide-mounted FFPE tissue sections were placed in a 4 slide holder. The slides were incubated three times in xylene for 3 min followed by 100% (v/v) ethanol twice for 3 min. The tissue was then hydrated twice in 85% (v/v) ethanol for 3 min, 70% (v/v) ethanol for 3 min, and distilled water for 3 min. The tissue was then blotted and scraped off the slide into a screw cap microfuge tube. To each sample tube (containing three FFPE 10 µm tissue sections), extraction buffer (0.2% RapiGest in 50 mM ammonium bicarbonate, NH_4_HCO_3_) was added and incubated at 95°C for 30 minutes with mixing at 1000 rpm (Thermomixer, Eppendorf, Enfield, CT). The samples were then cooled on ice for 5 minutes and sonicated twice in a cup horn probe (filled with ice water) at 50% power for 30 sec. The samples were then incubated at 80°C for 120 minutes with mixing at 1000 rpm and then cooled on ice for 5 min. 100 μL of 50 mM NH_4_HCO_3_, pH 8.0 was added, and the samples were sonicated twice in the cup horn probe (filled with ice water) at 50% power for 30 sec. Following processing, all samples were stored at -80°C until the day of digestion.

Protein concentrations of lysates were measured in triplicate using Micro BCA Protein Assay Kit (Pierce, #23235).

### 2.5 Protein Digestion

A mix of cleavable stable isotope-labeled peptide standards was added to the lysate at 200 fmol/capture. 500 μg frozen tissue lysates or 15-270 μg of protein (FFPE) was transferred to a deep-well plate for processing on an EpMotion 5057 (Eppendorf). Lysates were reduced in 30 mM TCEP for 30 minutes at 37°C with shaking, followed by alkylation with 50 mM IAM at room temperature. Lysates were then diluted with 0.8 mL 200 mM TRIS before Lys-C endopeptidase was added at a 1:50 enzyme:substrate ratio by mass and incubated for 2 hours at 37°C with mixing at 600 rpm (Thermomixer, EpMotion 5057). After 2 hours, trypsin was added at a 1:50 enzyme:substrate ratio. Digestion was carried out overnight at 37°C with mixing at 600 rpm. After 16 hours, the reaction was quenched with formic acid (final concentration 1% by volume).

Plasma (2 x 50 μL aliquots of each sample) was denatured with 150 μL of lysis buffer (25 mM Tris, 6 M Urea, 1 mM EDTA, 1 mM EGTA, 1% (v/v) Sigma protease inhibitor (#P8340), 1% (v/v) Sigma phosphatase inhibitor cocktail 2 (#P5726), 1% (v/v) Sigma phosphatase inhibitor cocktail 3 (#P0044)). The plasma was reduced with 30 mM TCEP at 37°C for 30 min, and alkylated with 50 mM IAM at room temperature for 30 min. Urea concentration was diluted 10-fold with 200 mM Tris prior to overnight digestion at 37°C with Lys-C/trypsin using a 1:50 (w/w) enzyme:substrate. Digestions were terminated with formic acid.

The mixture was desalted using Oasis HLB 96-well plates (Waters #WAT058951) and a positive pressure manifold (Waters #186005521) according to the following procedure: wash cartridge with 4 × 400 μL of 50% acetonitrile in 0.1% formic acid, equilibrate with 4 × 400 μL of 0.1% formic acid, load total volume of digest, wash with 4 × 400 μL of 0.1% formic acid, and elute with 3 × 400 μL of 50% acetonitrile in 0.1% formic acid. The eluates were lyophilized and stored at -80°C.

### 2.6 Peptide Immunoaffinity Enrichment

Enrichment was performed as previously described (49) with the following modifications. The final assay consisted of a mixture of 50 antibodies. Antibodies were crosslinked on protein G beads (GE Sepharose, #28-9513-79), and peptide enrichment was performed using 1 μg antibody - protein G magnetic beads for each target. Trypsin-digested lysate was resuspended in 200 μL 1X PBS + 0.01% CHAPS (pH was adjusted to 7.0 with 10 μL of 1 M Tris, pH 9). For plasma, the two digestion aliquots were combined after resuspension to a total volume of 200 μL. Beads were mixed in the incubation plate, washed twice in 1X PBS buffer + 0.01% CHAPS, washed once in 1/10X PBS + 0.01% CHAPS, and peptides were eluted in 26 μL of 5% acetic acid/3% acetonitrile/50 mM citrate. The elution plate was covered with adhesive foil and frozen at -80°C until analysis.

### 2.7 Liquid Chromatography Multiple Reaction Monitoring-Mass Spectrometry

LC-MS was performed with an Eksigent 425 nanoLC system with a nano autosampler and chipFLEX system (Eksigent Technologies, Dublin, CA) coupled to a 5500 QTRAP mass spectrometer (SCIEX, Foster City, CA). Peptides were loaded on a trap chip column (Reprosil C18-AQ, 0.5 mm x 200 μm, SCIEX, #804-00016) at 5 μL/min for 3 minutes using mobile phase A (0.1% formic acid in water). The LC gradient was delivered at 300 nL/minute and consisted of a linear gradient of mobile phase B (90% acetonitrile and 0.1% formic acid in water) developed from 2-14% B in 1 minute, 14-34% B in 20 minutes, 34-90% B in 2 minutes, and re-equilibration at 2% B on a 15 cm x 75 μm chip column (ChromXP 3C18-CL particles, 3 μm, SCIEX, #804-00001). The nano electrospray interface was operated in the positive ion MRM mode. Parameters for declustering potential (DP) and collision energy (CE) were taken from optimized values in Skyline. Scheduled MRM transitions used a retention time window of 210 seconds and a desired cycle time of 1.5 seconds, enabling sufficient points across a peak for quantification. A minimum of two transitions per peptide, including endogenous and spiked heavy peptides, were recorded for each light and heavy peptide.

### 2.8 Data Analysis

MRM data acquired on the 5500 QTRAP were analyzed by Skyline (50, 51). Peak integrations were reviewed manually, and transitions from analyte peptides were confirmed by the same retention times and relative transition areas of the light peptides and heavy stable isotope-labeled peptides. Transitions with detected interferences were not used in the data analysis. Integrated raw peak areas were exported from Skyline and total intensity was calculated using Peak Area + Background. Transitions were summed for each light/heavy pair and peak area ratios were obtained by dividing peak areas of light peptides by that of the corresponding heavy peptides (or vice versa for response curves). All measurements were filtered by the LLOQ (i.e., all measurements were required to be above the LLOQ). Peak area ratios were log (base 2) transformed for statistical analysis.

Quantitative RNA sequencing data (FPKM) was available for 74/110 frozen tissues (52). MRM peak area ratios were log (2) transformed and correlated with gene expression data using Pearson regression. Correlations were done within each tumor type and across all tumor types (n = 74).

Minimum tissue requirements were calculated by using frozen tissue results of signal-to-noise levels measured at 500 μg input. The signal-to-noise levels were compared to the LOQ for each analyte using a one-sample t-test and those analytes with 95% confidence above LOQ were further converted to input mass using linear scale dilution. The confidence interval in the minimum tissue calculation was determined based on the standard deviation of signal-to-noise distribution and degrees of freedom (equal to the number of samples for each tissue site -1).

### 2.9 Fit-For-Purpose Assay Validation

Four experiments (described below) were performed to characterize the analytical performance of the assays: i) digestion time course for stable recovery of peptides, ii) response curves, iii) repeatability, and iv) stability.

#### 2.9.1 Digestion Process Time Course

Recovery of peptides was measured by a time course study using triplicate process replicates of 150 μg of protein of a pooled lysate from cell lines processed according to the trypsin digestion workflow described above. Aliquots were prepared for quenching of the digestion at four time points (2, 6, 16, and 24 hours). Digested samples were enriched and analyzed as described above.

#### 2.9.2 Response Curve

Response curves were generated in a background matrix consisting of an equal mixture of protein lysate from 5 commercially obtained (FFPE) lung tumors, an equal mixture of lysates from 4 frozen tumors, or commercially obtained plasma. Aliquots (100 μg of tissue, 10 μL plasma) of the matrix were spiked with cleavable heavy stable isotope-labeled peptides covering the concentrations 200, 20, 2, 0.8, 0.32, 0.128, 0.0512, and 0.02048 fmol/uL for plasma lysates and 20,000, 2,000, 80, 32, 12.8, 5.12, and 2.048 fmol/mg for FFPE and frozen tissue lysates. Concentrations for spikes for heavy extended peptides corresponding to ADAM17.VDNE, CD163.LVDG, GAPDH.GALQ, TNFRSF9.NQIC, and VSIR.GHDV were ten times higher. Light peptide was also spiked into the digested lysate at 20 fmol/uL for plasma and 2,000 fmol/mg for FFPE and frozen lysates. Blanks were prepared using background matrix with light peptide (no heavy spike). All points were analyzed in four replicates. Curves were analyzed using Skyline by performing linear regression using log transformed data on all points above the lower limit of quantification. The Lower Limit of Quantifications (LLOQs) were obtained by empirically finding the lowest point on the curve that had CV <20% in the curve replicates. The upper limit of quantification (ULOQ) was determined by the highest concentration point of the response curve that was maintained in the linear range of the response. For curves that maintained linearity at the highest concentration measured, the ULOQ is a minimum estimate.

#### 2.9.3 Repeatability

Repeatability was determined using the same matrix used to generate the response curves except 50 mM ammonium bicarbonate was used in place or Tris for the protein digestion. Cleavable heavy peptide standards were spiked at three levels (low, medium, and high) at 1.33e2, 1.33e3, 1.33e4 fmol/mg for FFPE samples and 2, 20, 200 fmol/uL for plasma sample. Spikes for heavy extended peptides corresponding to ADAM17.VDNE, CD163.LVDG, GAPDH.GALQ, TNFRSF9.NQIC, and VSIR.GHDV were ten times higher at 1.33e3, 1.33e4, 1.33e5 fmol/mg for FFPE samples and 20, 200, 2,00 fmol/uL for plasma samples. Analogous to response curve experiments, following digestion, light peptides were also spiked at 20 fmol/uL for plasma and 1,333.3 fmol/mg for FFPE lysates. Complete process triplicates (including digestion, capture, and mass spectrometry) were prepared and analyzed on five independent days. Intra-assay variation was calculated as the mean CV obtained within each day. Inter-assay variation was the CV calculated from the mean values of the five days.

#### 2.9.4 Peptide Stability

Stability of the enriched peptides was determined by analyzing aliquots of the medium spike level sample used in repeatability studies after storage at 4°C in the autosampler for approximately 8 hours, storage at 4°C for 48 hours, after 2 freeze-thaw cycles, and after extended storage at -80°C for 5 weeks. Each condition was measured in process triplicate.

### 2.10 Characterization of Antibodies in Immunoblotting and Protein Array

Recombinant proteins and over-expressed lysates were obtained from Origene (Rockville, MD) or Novus Biologicals (Littleton, CO), see **Supplementary Table 1** for catalog numbers. Western immunoassays were performed using traditional immunoblotting techniques according to the designed protocol described in the CPTAC Antibody Portal (antibodies.cancer.gov) SOP M-103 with the following modifications. Proper sample loading on the 4-20% Criterion TGX Stain-free precast gels (Bio-Rad, # 5678094) was verified by rapid florescence detection with ChemiDoc MP imager. Traditional immunoblotting used 10 μg/mL recombinant protein under reducing conditions (20 mL, 200 ng total protein/lane). Whole cell lysates were diluted to 2.5 mg/mL in reducing conditions (20 mL, 50 μg total protein/lane). Transfers of protein from Bio-Rad precast gels were performed by Bio-Rad Turbo-Blot at “High MW” setting for 10 minutes. Blocking of the membrane was performed using Bio-Rad Blotting Grade Blocker (Bio-Rad, #1706404) at 5% in 1X PBS/0.5% Tween-20. Primary antibodies (1 mg/mL) were diluted in 1X PBS/0.5% Tween-20 to a dilution of 1:5000 at a total volume of 25 mL. Washing of membrane was conducted using 1X PBS/0.5% Tween-20 three times. Secondary HRP-linked rabbit specific antibody (Jackson ImmunoResearch, West Grove, PA, 111-035-144) or secondary HRP-linked mouse antibody (Jackson Immunoresearch Laboratories 115-035-062) was diluted at 1:5000 in 1 X PBS/0.5% Tween-20 at a final volume of 25 mL. Immuno-detection was performed using colorimetric substrate Opti-4-CN (Bio-Rad, #1708235) at 1 mL per blot or using enhanced chemiluminescence using Clarity Western ECL Substrate (BioRad, #1705061) at 1 mL per blot. Development of immunoblot was captured using Bio-Rad ChemiDoc MP imaging system.

The Simple Western (Wes, ProteinSimple, San Jose, CA, #004-600) system was used to detect primary antibody binding to a target protein in cell lysates (MCF10A, LCL57). The Simple Westerns were performed following the procedures detailed in the CPTAC Antibody Portal (antibodies.cancer.gov) SOP M-134 with the following modifications. Cell lysates were run using the 12-230 kDa separation module, 8 x 25 capillary cartridges (ProteinSimple, #SM-W004), and detected with Anti-Rabbit Detection Module (ProteinSimple, #DM-001) or Anti-mouse Detection Module (ProteinSimple, #DM-002). Cell lysates were run at a concentration of 200 μg/mL and incubated with primary antibodies diluted 1:500.

Protein array analyses were performed using NCI-60 cell lines obtained from the Cancer Research Technology Program at NCI-Frederick (Frederick, MD). The NCI-60 cell lines were collected at the log phase growth and protein prepared by resuspending cell pellets in RIPA (Thermo Fisher Scientific, #89900) per the manufacturer’s recommendations; total protein concentration was measured by BCA Protein Assay kit (Thermo Fisher Scientific, #23225). Quantification of protein expression values was performed by well-based reverse phase protein array (RPPA) as previously reported (53, 54). Briefly, five microliters (100 ng/well) of NCI-60 cell line antigens in PBST (1X PBS, 0.1% Tween-20) were applied onto 96-well Multi-Array™ plates (96 HB SECTOR Plate, Meso Scale Discovery, Gaithersburg, MD). The plates were allowed to dry at room temperature for 2 hours. Prior to primary antibody incubation, the antigen-coated plates were blocked with 5% non-fat dry milk in PBST for 1 hour at room temperature. Target-specific antibodies were diluted (1:1000 to 1:5000) with 5% BSA in PBST. For each cell line, 25 μL of antibody were added and incubated overnight at 4 °C. The plates were washed with PBST and followed by a 90 min incubation with goat anti-mouse or anti-rabbit SULFO-TAG™ antibodies (Meso Scale Discovery) at a dilution of 1:2000 (0.5 µg/mL) containing 5% non-fat dry milk in PBST. Plates were washed, and MSD-T read buffer was added to the plate to detect binding signals using MESO QuickPlex SQ 120 reader (Meso Scale Discovery). PBST-coated wells were included on each plate as a control of non-specific binding. For each mAb, the electrochemical luminescence value of each cell line was normalized by the average value of the 60 cell lines. After normalization, levels below 0.5 were interpreted as weak or negative expression and levels above 1.5 were interpreted as strong, positive expression.

### 2.11 Public Availability of Data and Reagents

Targeted mass spectrometry data are available in the supplementary tables and Panorama Public (55), a public repository of targeted proteomics experiments (https://panoramaweb.org/IO1immunoMRM.url. Characterization data for assays can be found *via* the CPTAC Assay Portal (https://assays.cancer.gov) and antibodies are available through the CPTAC Antibody Portal (antibodies.cancer.gov) (see **Supplementary Table 2** for IDs).

## 3 Results

### 3.1 Development of a Targeted, Multiplexed IO-1 Immuno-MRM Assay

Because there are no adequate predictive or prognostic biomarkers to guide delivery of immunotherapies, there is great interest in high quality assays to quantify immunomodulatory proteins for correlative studies in clinical trials in hopes of identifying single or panels of biomarkers showing clinical utility (56). Proteins and phosphorylation sites related to immunomodulatory functions were identified as targets for assay development through literature searching and expert consultation. A panel of experts in immuno-oncology was convened from academia and industry to nominate immunomodulatory proteins of interest for assay development (**Table 1**) to support correlative studies to identify novel biomarkers or response or immune-related adverse events. Stoichiometric surrogate peptides for the target proteins were identified using established procedures (88, 89), by mining liquid chromatography-tandem mass spectrometry (LC-MS/MS) proteomic and phosphoproteomic datasets (16, 17, 90–98) and public databases (52, 99, 100) for empirical evidence of LC-MS/MS detectability. Peptides were ranked based on the intensity and frequency of empirical observations in the datasets, chemical properties (e.g., amino acid composition, hydrophobicity), and physical properties (e.g., length, position in protein). Finally, peptides with frequent mutation sites [contained in dbSNP (101)] and PTMs [PhosphoSitePlus (102)] were avoided.

**Table 1:** Peptides targeted for the multiplexed IO-1 immuno-MRM assay panel.

Gene Symbol	Peptide Modified Sequence	Acc. ID	Description	Role in Immuno-Oncology	Reference
ADAM17	VDNEELLPK	P78536	ADAM metallopeptidase domain 17	Processing of inflammatory agents	(57)
ANXA1	AAYLQETGKPLDETLK	P04083	annexin A1	Innate immune response	(58)
ANXA1	GVDEATIIDILTK	P04083	annexin A1	Innate immune response	(58)
ARG2	TFDLLIGK	P78540	arginase 2	Arginine metabolism is a regulator of immune response	(59)
ATM	NLS[ph]DIDQSFNK	Q13315	ATM serine/threonine kinase	Immunoglobulin class switch recombination	(60)
ATM	NLSDIDQSFNK	Q13315	ATM serine/threonine kinase	Immunoglobulin class switch recombination	(60)
ATM	SLEIS[ph]QSYTTTQR	Q13315	ATM serine/threonine kinase	Immunoglobulin class switch recombination	(60)
ATM	SLEISQSYTTTQR	Q13315	ATM serine/threonine kinase	Immunoglobulin class switch recombination	(60)
CCL5	EYFYTSGK	P13501	C-C motif chemokine ligand 5	Trafficking of T cells to tumors	(9)
CD14	FPAIQNLALR	P08571	CD14 molecule	Innate immune response	(61)
CD163	LVDGVTEC[cam]SGR	Q86VB7	CD163 molecule	Macrophage activation	(62)
CD274	NIIQFVHGEEDLK	Q9NZQ7	CD274 molecule	Killing of cancer cells	(9)
CD33	ILIPGTLEPGHSK	P20138	CD33 molecule	Inflammatory response	(63)
CD40	SC[cam]SPGFGVK	P25942	CD40 molecule	Cancer antigen presentation	(9)
CD40	YC[cam]DPNLGLR	P25942	CD40 molecule	Cancer antigen presentation	(9)
CD47	STVPTDFSSAK	Q08722	CD47 molecule	Macrophage activation	(62)
CD70	LYWQGGPALGR	P32970	CD70 molecule	Priming and activation	(9)
CD74	C[cam]QEEVSHIPAVHPGSFRPK	P04233	CD74 molecule	Antigen processing	(64)
CEACAM8	IIGYVISNQQITPGPAYSNR	P31997	CEA cell adhesion molecule 8	Modulation of immune cell activity	(65)
CX3CL1	ALGTSPELPTGVTGSSGTR	P78423	C-X3-C motif chemokine ligand 1	Trafficking of T cells to tumors	(9)
CXCL10	VEIIATMK	P02778	C-X-C motif chemokine ligand 10	Trafficking of T cells to tumors	(9)
CXCL13	SIVC[cam]VDPQAEWIQR	O43927	C-X-C motif chemokine ligand 13	Trafficking of B cells	(66)
ENTPD1	SLSNYPFDFQGAR	P49961	ectonucleoside triphosphate diphosphohydrolase 1	Immune system suppression	(67)
FAS	EAY[ph]DTLIK	P25445	Fas cell surface death receptor	Immune cell survival, differentiation, and activity	(68)
FAS	EAYDTLIK	P25445	Fas cell surface death receptor	Immune cell survival, differentiation, and activity	(68)
GAPDH	GALQNIIPASTGAAK	P04406	glyceraldehyde-3-phosphate dehydrogenase	Potential control protein	(69)
HAVCR2	GAC[cam]PVFEC[cam]GNVVLR	Q8TDQ0	hepatitis A virus cellular receptor 2	Killing of cancer cells	(9)
ICAM1	DGTFPLPIGESVTVTR	P05362	intercellular adhesion molecule 1	Infiltration of T cells into tumors	(9)
ICOSLG	GLYDVVSVLR	O75144	inducible T cell costimulator ligand	Stimulation of T cells	(70)
IL18	ISTLSC[cam]ENK	Q14116	interleukin 18	Immune response regulator	(71)
ITGAE	VSYQLQTPEGQTDHPQPILDR	P38570	integrin subunit alpha E	Trafficking of T cells	(72)
LGALS1	SFVLNLGK	P09382	galectin 1	Regulator of T cell apoptosis	(73)
LIME1	SSTC[cam]GAGTPPASSC[cam]PSLGR	Q9H400	Lck interacting transmembrane adaptor 1	Immune cell signaling	(74)
LIME1	SSTC[cam]GAGT[ph]PPASSC[cam]PSLGR	Q9H400	Lck interacting transmembrane adaptor 1	Immune cell signaling	(74)
NFKB2	IEVDLVTHSDPPR	Q00653	nuclear factor kappa B subunit 2	Transcription factor related to immunity	(75)
NT5E	VIYPAVEGR	P21589	5’-nucleotidase ecto	Maintenance of immune cells	(76)
PDCD1LG2	ATLLEEQLPLGK	Q9BQ51	programmed cell death 1 ligand 2	Essential for T cell proliferation	(77)
PECAM1	DQNFVILEFPVEEQDR	P16284	platelet and endothelial cell adhesion molecule 1	Leukocyte transendothelial migration	(78)
PSMA1	ETLPAEQDLTTK	P25786	proteasome 20S subunit alpha 1	Antigen processing	(79)
PTGS2	ALPPVPDDC[cam]PTPLGVK	P35354	prostaglandin-endoperoxide synthase 2	Immune response regulator	(80)
PTPRC	DPPSEPSPLEAEFQR	P08575	protein tyrosine phosphatase receptor type C	T cell regulator	(81)
PTPRC	LFLAEFQSIPR	P08575	protein tyrosine phosphatase receptor type C	T cell regulator	(81)
RIF1	ASQGLLSSIENSESDSSEAK	Q5UIP0	replication timing regulatory factor 1	Required for immunoglobulin class switch recombination	(82)
STAT1	YTYEHDPITK	P42224	signal transducer and activator of transcription 1	Regulator of tumor cell immune evasion	(83)
STAT3	TGVQFTTK	P40763	signal transducer and activator of transcription 3	Regulator of tumor cell immune evasion	(83)
STAT6	GYVPATIK	P42226	signal transducer and activator of transcription 6	Regulator of tumor cell immune evasion	(83)
TAP2	EAVGGLQTVR	Q03519	transporter 2, ATP binding cassette subfamily B member	Antigen processing	(84)
TNFRSF14	EDEYPVGSEC[cam]C[cam]PK	Q92956	TNF receptor superfamily member 14	Immune cell survival, differentiation, and activity	(68)
TNFRSF17	SLPAALS[ph]ATEIEK	Q02223	TNF receptor superfamily member 17	Immune cell survival, differentiation, and activity	(68)
TNFRSF17	SLPAALSATEIEK	Q02223	TNF receptor superfamily member 17	Immune cell survival, differentiation, and activity	(68)
TNFRSF9	NQIC[cam]SPC[cam]PPNSFSSAGGQR	Q07011	TNF receptor superfamily member 9	Immune cell survival, differentiation, and activity	(68)
TNFSF9	EGPELSPDDPAGLLDLR	P41273	TNF superfamily member 9	Immune cell survival, differentiation, and activity	(68)
VCAM1	TQIDSPLSGK	P19320	vascular cell adhesion molecule 1	Inflammatory response	(85)
VSIR	GHDVTFYK	Q9H7M9	V-set immunoregulatory receptor	Negative regulator of T cell function	(86)
VTCN1	EGVLGLVHEFK	Q7Z7D3	V-set domain containing T cell activation inhibitor 1	T cell regulator	(87)

Modifications of “[ph]” indicate phosphorylation of the preceding Ser, Thr, or Tyr, and “[cam]” indicates carbamidomethylated Cys.

A list of 55 peptides (including multiple peptides per protein) were selected for assay development, representing 46 proteins and including 5 phosphorylation sites. Peptide sequences selected for assay development are listed in **Table 1**. Unlabeled (i.e., light) tryptic peptides were synthesized for assay development, and cleavable heavy stable isotope-labeled peptides were synthesized for use as internal standards. The cleavable standards incorporate 2-5 additional amino acids (corresponding to the native protein sequence) on either side of the tryptic cut sites. This strategy was adopted in order to spike the standards into the sample prior to digestion and provide a within-sample control for trypsin digestion (20). The synthetic light peptides were used to optimize the selection of specific precursor and fragment ion pairs (i.e., transitions), determining the chromatographic retention time of each peptide, and optimizing collision energy parameters in the mass spectrometer (**Supplementary Table 3**).

**Figure 1:**
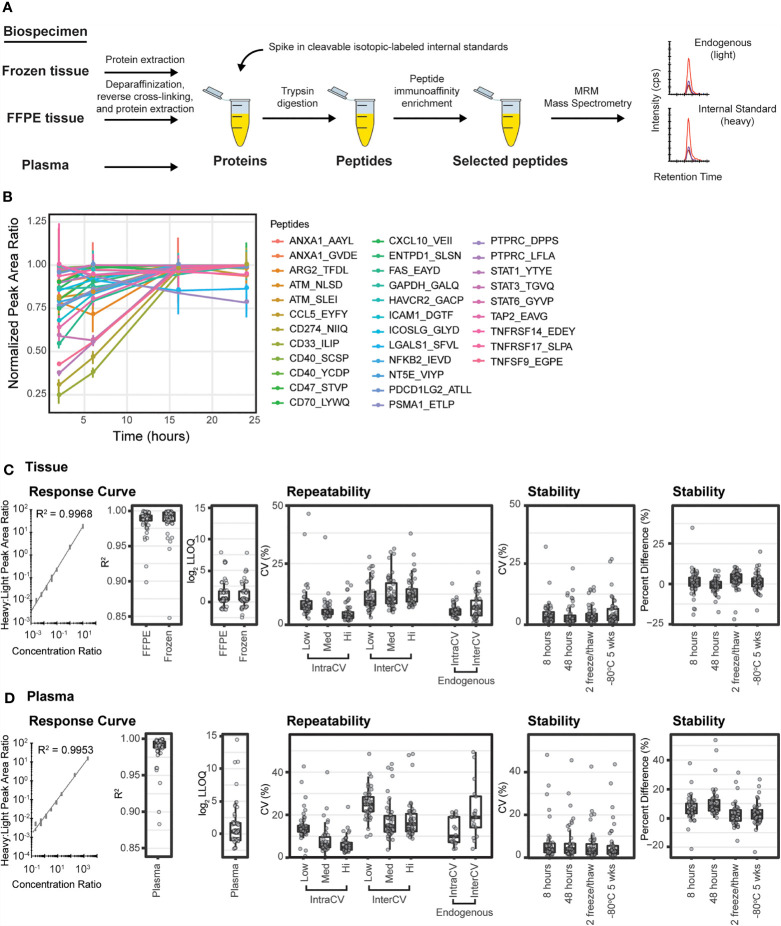
Immuno-MRM enables highly multiplexed protein quantification. **(A)** The immuno-MRM assay workflow commences with generation of a protein lysate from the biospecimen of interest. Cleavable stable isotope labeled standards unique to each targeted peptide sequence are spiked into the sample at a known concentration. The protein mixture is converted to peptides by enzymatic digestion (Lys-C and trypsin). Custom monoclonal antibodies coupled to magnetic beads are used to enrich the endogenous peptides and labeled standards. The eluate is analyzed by multiple reaction monitoring-mass spectrometry, where analyte peptides and internal standards coelute with equivalent relative areas of monitored transitions. High sensitivity is achieved through analyte enrichment and optimization of mass spectrometer parameters for the targeted peptides. High specificity is maintained through optimal selection of fragment ion transitions. **(B)** Trypsin-mediated release of peptides was produced by overnight enzymatic digestion of proteins from a pool of cell lysates. The peak area ratios (light:heavy) for the 33 endogenous peptides detected were normalized to the maximum timepoint and plotted over time. Error bars are the standard deviation of three replicates. **(C)** Performance figures of merit for assays characterized in tissue matrix. **(D)** Performance figures of merit for assays characterized in plasma matrix. A representative response curve for the peptide VEIIATMK from CXCL10. Each concentration point was measured in triplicate. Distribution of R^2^ values from linear regression of the response curves. Distribution of lower limit of quantification (LOQ) where each point refers to concentration determined by the lowest point on the curve with less than 20% CV. Repeatability is characterized by the distribution of CV values for intra- (within day) and inter- (between day) variability at three concentrations, in addition to the measurement of endogenous peptide. Each point corresponds to the average %CV for a peptide measured at three concentrations, Low (Lo), Medium (Med), and High (Hi) in triplicate over five days (n=15 at each concentration for a peptide). Endogenous measurements refer to the intra (within day) and inter- (between day) variability of endogenous peptides detected above the LOQ in five replicates measured over 5 days (n = 25). Stability shows the distribution of %CV and relative percent difference for 4 conditions compared to immediate analysis: (i) stored at 8 hours at 4°C, (ii) 48 hours at 4°C, (iii) after two freeze-thaw cycles, and (iv) stored at -80°C for 5 weeks. For box plots, the line shows the median value, boxes show the inner quartiles, and the whiskers show 5-95% of data.

Immuno-MRM uses anti-peptide antibodies (103) for enrichment prior to LC-MRM and is applicable for quantifying expression of high and low abundance proteins as well as phosphopeptides (**Figure 1A**). For the immuno-MRM assay panel, we generated custom anti-peptide monoclonal antibodies to the linear peptide sequences using established approaches (25, 26). We used 50 custom monoclonal anti-peptide antibodies (**Supplementary Table 2**) for the multiplexed immuno-MRM assay, including 35 antibodies generated for this study and 15 antibodies from previously characterized assays (26, 40).

### 3.2 Fit-For-Purpose Method Validation of the IO-1 Immuno-MRM Assay

Trypsin digestion has been identified as a source of variability in sample processing for targeted proteomics (29, 104). Therefore, to achieve reproducible digestion, we sought to identify a timepoint where release of the tryptic peptides (light and heavy) was most stable and at a maximum. Recovery of peptides following trypsin digestion was characterized using a time course digestion study using an optimized procedure incorporating Lys-C and trypsin for digestion (105). Aliquots of pooled cell line lysates were spiked with heavy peptides and digested according to the methods described herein. To evaluate the release of endogenous tryptic peptides over time, aliquots were taken after 1, 6, 16, and 24 hours of incubation with trypsin and subjected to immunoaffinity enrichment and analysis by LC-MRM. The recovery of light peptides reached a stable maximum at the 16-hour timepoint, with two peptides showing slightly lower (~20%) signals (**Figure 1B**). To achieve the maximum recovery for most peptides in the assay, we used overnight (i.e., 16 hours) digestion for processing samples.

Performance of the multiplexed IO-1 immuno-MRM assay panel was characterized in tissue and plasma matrices according to published guidelines (47, 106) to establish figures of merit including linearity, limits of quantification, repeatability, and stability. The linear ranges and limits of quantification (LOQs) were determined by response curves using pooled background matrices of protein lysates from i) frozen lung tumor tissues, ii) formalin fixed and paraffin embedded (FFPE) lung tumor tissues, and iii) plasma. Aliquots (150 µg) of the pooled tissue lysates or 10 µL aliquots of plasma were spiked with synthetic cleavable heavy stable isotope-labeled standards and serially diluted to yield concentrations of 2000, 200, 20, 8, 3.2, 1.28, 0.512, 0.205, and 0 (blank) fmol/mg or fmol/µL, respectively, and each concentration level was digested in process triplicate. Synthetic light tryptic peptides were added at a constant concentration of 200 fmol/mg or 200 fmol/µL prior to desalting the digested peptides. The monoclonal antibodies were coupled to magnetic beads and used to enrich the peptides. The eluates were analyzed by LC-MRM-MS. Peptide specificity was confirmed by equivalent retention times and relative transition areas of the heavy and light peptides.

Peak area ratios (heavy:light) were plotted as a function of analyte concentration to determine the assay figures of merit in the response curves. **Figures 1C, D** show representative response curves measured in tissue and plasma. Lower limits of quantification (LLOQs) were determined by the lowest point with CV < 20%. Linear ranges were determined by using points on the linear regression with correlation coefficients greater than 0.9. For assays where the highest concentration point was still linear, the linear range was a minimum estimate. Figures of merit are reported for each peptide in **Supplementary Table 4**. Median linear dynamic range was ≥3.2 orders of magnitude in all matrices with median LLOQ 12.8 fmol/mg (range 2-2000 fmol/mg) in tissues and 0.128 fmol/µL (range 0.02-2000 fmol/µL) in plasma. The characterized LLOQs and linear ranges were the same for frozen and FFPE tissues (**Supplementary Figure 1**). Three peptides (LIME1.SSTC[cam]GAGT[ph]PPASSC[cam]PSLGR, LIME1.SSTC[cam]GAGTPPASSC[cam]PSLGR, and VSIR.GHDVTFYK) exhibited low signal in the curves, likely due to poor antibody activity when cross-linked to the beads, and failed to produce adequate linearity for further use.

Intra-assay (within day) and inter-assay (between day) repeatability were determined by performing complete process triplicate measurements for the multiplexed assay at three concentrations of spiked peptides over 5 days. Heavy cleavable peptide standards were spiked into 150 µg aliquots of the pooled FFPE tissue lysate or 10 µL aliquots of plasma matrix at three concentrations (10, 100, 1000 fmol/mg; low, medium, high) with addition of equal amounts of light peptides (200 fmol/mg) to each aliquot. Specificity was confirmed using the same criteria as described above. For the assays measured in tissue matrix, the median intra-assay variability was 7.5%, 5.4%, 3.5% for low to high concentration samples and the median inter-assay variability was 9.5%, 10.8% and 11.8% (low to high) (**Figure 1C** and **Supplementary Table 5**). Two outlier assays (ATM.NLS[ph]DIDQSFNK and ATM.NLSDIDQSFNK) showed high intra- or inter-assay variability in tissue samples due to signal intensities below the LOQ at the lowest concentration values. For the assays measured in plasma matrix, the median intra-assay variability was 13.6%, 5.8%, 4.5% from low to high concentration samples and the median inter-assay variability was 24.9%, 14.9% and 16.5% from low to high concentration samples (**Figure 1D** and **Supplementary Table 5**). Three assays (ANXA1.AAYLQETGKPLDETLK, PTPRC.LFLAEFQSIPR, PSMA1.ETLPAEQDLTTK) showed high variability when applied to plasma. High variability was likely due to different factors for the peptides. ANXA1.AAYL had high variability in the heavy signal, PTPRC.LFLA was likely due to instability of the peptide following processing (see below), and PSMA1.ETLP had one day with outlier values compared to other days.

To characterize expected variability in measuring endogenous proteins, heavy cleavable peptide standards were spiked into 150 µg aliquots of a pooled FFPE lung tissue lysate and 10 µL aliquots of plasma. Measurements were made using 5 complete process replicates of endogenous peptide over 5 days (n=25) (**Figures 1C, D**). Forty-one peptides were detected above LLOQ in the endogenous tissue sample. The median intra-assay variability for endogenous detection was 5.2% (range 2.4-16.9%) and the median inter-assay variability was 6.9% (range 0.8-21.4%). Twenty peptides were detected above LOQ in the endogenous plasma sample. The median intra-assay variability was 12.2% (range 4.1-34%) and the median inter-assay variability was 21% (range 4.5-62%).

Peptide stability was evaluated by spiking heavy peptide (200 fmol/mg) into 150 µg aliquots of the pooled FFPE tissue lysate or 200 fmol/µL into 10 µL aliquots of plasma matrix, followed by digestion and immunoaffinity capture. The samples were analyzed after storage under four conditions: (i) 4°C on the autosampler for 8 h, (ii) 4°C on the autosampler for 48 h, (iii) after two freeze-thaw cycles, and (iv) -80°C for 5 weeks; control samples were analyzed immediately. Each test case was measured in triplicate. The variability (%CV) and percent difference, comparing peak area ratio between control samples and samples with different handling conditions, were used to evaluate peptide stability. The median %CV for the test samples ranged between 2.5-8.5% in FFPE and 3.1-6.5% in plasma (**Supplementary Table 6**), within expectations based on repeatability measurements (**Supplementary Table 5**) for both matrices. The median percent difference relative to the fresh sample ranged 0.1-3.5% in FFPE and 2.0-5.6% in plasma, indicating acceptable stability for the peptides (**Figures 1C, D** and **Supplementary Table 6**). In FFPE, three assays (CCL5.EYFYTSGK, ATM.NLS[ph]DIDQSFNK, CD274.NIIQFVHGEEDLK) showed high variability and/or relative difference, indicating these peptides should not be allowed to sit on the autosampler for longer than 8 hours. Likewise, three assays (LGALS1.SFVLNLGK, CD274.NIIQFVHGEEDLK, PTPRC.LFLAEFQSIPR) showed high variability or percent difference in plasma samples after storage on the autosampler, indicating these peptides should be analyzed by LC-MRM within 8 hours.

### 3.3 Determination of Assay Utility in Tissue and Sample Requirements

We next applied the multiplex assay to a panel of tumor tissue specimens with two aims: (i) evaluate the utility of the assay for measuring endogenous levels of analytes in clinical biospecimens, and (ii) determine sample requirements for analyte detection in tissue where clinical material may be limited. The tissue panel included 110 frozen and 25 FFPE biospecimens collected from 12 different tumor types.

**Figure 2:**
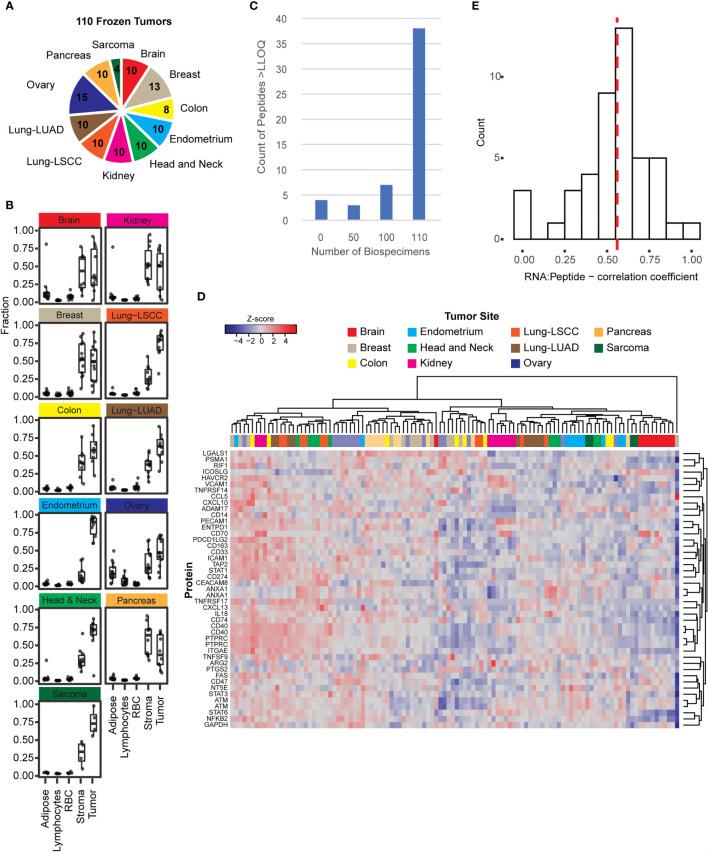
The immuno-MRM assays show detection in frozen tissues. **(A)** Frozen tissues were obtained for 110 tumors from 11 tumor types. The number of each type is indicated in the pie chart. **(B)** The relative fractions of adipose, lymphocytes, red blood cells, stroma, and tumor cells were plotted for tumors with available images (108 out of 110). Cellular microheterogeneity was determined by using the HALO algorithm. Each point represents an individual tumor. Box plots show median (line), inner quartiles (box), and 5-95% range (vertical lines). **(C)** Distribution of peptide detection plotted as a histogram, showing the number of peptides detected above LOQ across the 110 frozen tumors. **(D)** Heatmap showing unsupervised clustering of analytes detected above LOQ in > 50% of tumor specimens. Peak area ratios (light:heavy) were normalized for each peptide analyte, and the z-score was used for clustering. **(E)** Histogram showing correlation of protein expression measured by immuno-MRM with mRNA transcript level determined by RNAseq (52). The median is indicated by a dotted line (0.559).

Frozen tissue biospecimens were obtained from 11 tumor types including brain, breast, colorectal, endometrium, head and neck, kidney, lung (squamous cell carcinoma and adenocarcinoma), ovarian, pancreas, and soft tissue sarcoma (**Figure 2A**). The distribution of cellular heterogeneity was evaluated using the H&E-stained slides, and a supervised algorithm (40) (HALO) was used to assign the fraction of tissue area pertaining to predominant cell types (e.g., adipose, lymphocytes, red blood cells, stroma, and tumor). The distribution of these cellular subtypes is plotted in **Figure 2B** for the frozen tumors. Overall, most of the biospecimen area can be assigned to tumor and stromal cells, with small proportions of adipose, lymphocytes, and red blood cells. Notably, ovarian tumors contained the highest fraction of adipocytes. These fractions show that the tumors were relatively good quality specimens, with median percentage of tumor cells >50%.

To determine the ability of the IO-1 immuno-MRM assay to measure endogenous proteins, we used 500 µg aliquots of the frozen tissue lysates as input. Each biospecimen was independently digested, enriched for the IO-1 analytes, and analyzed by LC-MRM. Specificity of endogenous peptide detection was assured by equal retention times and relative areas of light and heavy transitions. Overall, 48/52 peptides corresponding to 45 proteins were detected above LLOQ in the frozen tissue biospecimens. Four peptides were not detected in the biospecimen panel: phospho-ATM pS367, phospho-ATM pS2996, phospho-FAS pY291, and unmodified TNFRSF9. Most peptides were detected in all samples, with a small number of peptides detected below the LLOQ in a subset of samples (see **Figure 2C**). The median inter-individual CV was 77% (range 32-676%). This variation is likely due to differences between the individual tumors and not differences in the tissue type, since unsupervised clustering of the expression levels for the peptides in the IO-1 multiplex panel (**Figure 2D**) did not separate the samples by location. This is not surprising, since these proteins were not chosen for site-specific differences in expression, but rather for their role in immunomodulatory functions, which may vary from tumor to tumor. Finally, correlation of expression levels with mRNA (17, 52, 90–97, 107) showed a median correlation coefficient of 0.56 (**Figure 2E**), consistent with previous reports measuring protein-RNA correlation (15–17).

**Figure 3:**
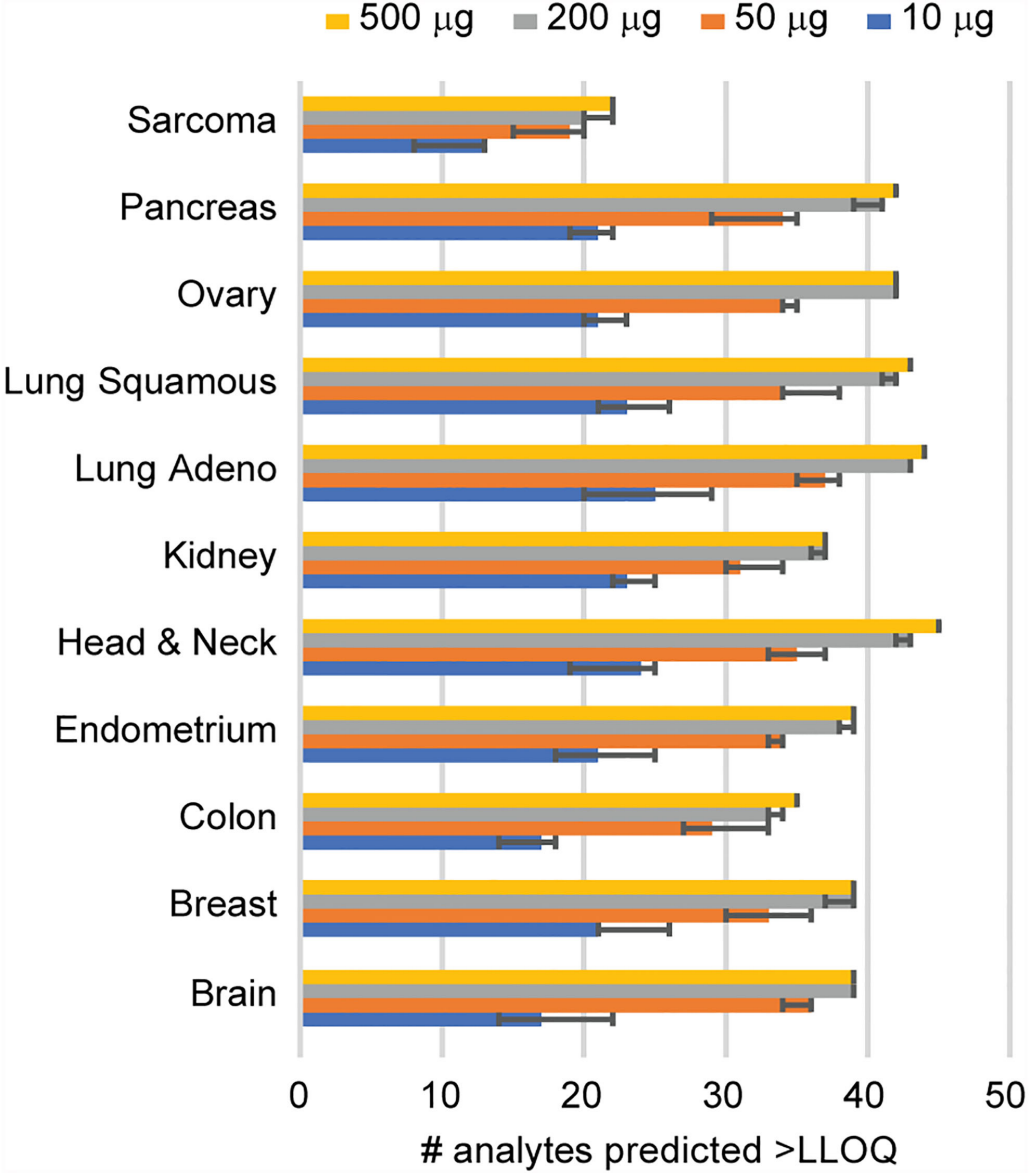
Determination of sample requirements for detection in tissue. The number of analytes predicted for decreasing amounts of tissue was determined from the signal-to-noise ratio measured using 500 μg protein digest input. Error bars show the 95% confidence interval.

Given that clinical biospecimens often have limited available material, we next sought to determine the sample requirements for detection of endogenous protein in tissue to use as a guide for expectations in future studies. We used the results of the frozen tissue array (**Figure 2**) to estimate the minimum amount of tissue needed for the analyte to remain above the LOQ using the distribution of signal-to-noise values measured in tumor samples, compared to the LLOQ characterized in tissue matrix. **Figure 3** shows the number of peptides predicted to be detected for tissue inputs ranging from 10-500 µg. Notably, the number of peptides detected as the amount of tissue decreases remains within ~80% of total, even with ten-fold less input. This indicates that the assays are amenable to a range of biospecimen sizes and input amounts.

To evaluate the potential for applying the assays to retrospectively collected biospecimens, we evaluated the detection of peptides in FFPE tissues representing 5 tumor types, including colorectal, head and neck, lung (squamous cell carcinoma and adenocarcinoma), and prostate (**Supplementary Figure 2**). Optimized sample processing conditions were used to process the samples (48), however the limited material available for these samples resulted in sample inputs ranging from 2 to over 10-fold less material compared to frozen tissue biopsies. Despite these limitations, the assay successfully quantified 33/52 peptides above LOQ in more than half of samples (**Supplementary Figure 2**).

### 3.4 The Multiplexed Assay Shows Utility for Measurements of Plasma Protein Expression

**Figure 4:**
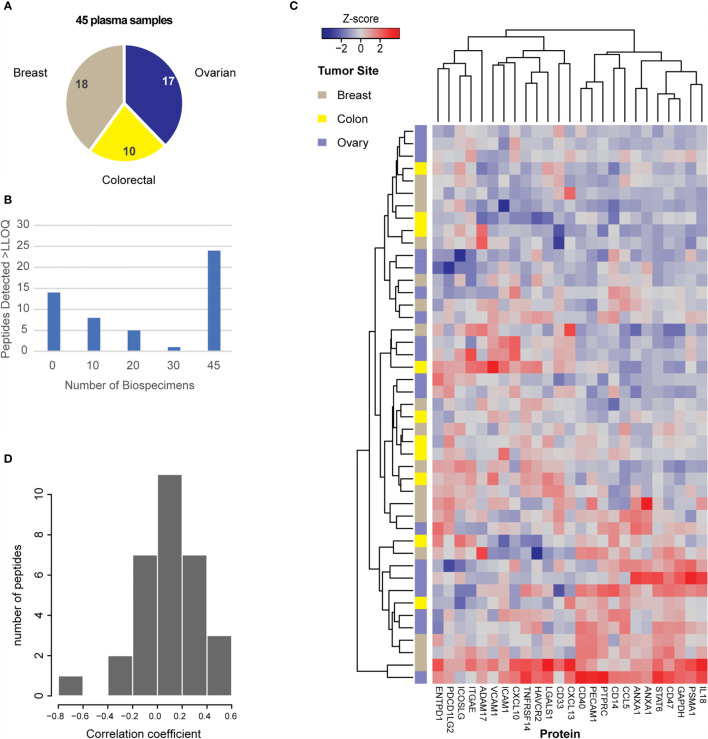
Utility of the assays for measurement of protein expression in plasma. **(A)** Plasma samples were obtained for 45 patients with breast, colorectal, or ovarian tumors, as indicated in the pie chart. **(B)** Distribution of peptide detection plotted as a histogram, showing the number of peptides detected above LOQ across the 45 plasmas using 100 μL aliquots of plasma as input. **(C)** Heatmap showing unsupervised clustering of analytes detected above LOQ in >50% of plasma samples. Peak area ratios (light:heavy) were normalized for each peptide analyte, and the z-score was used for clustering. **(D)** Histogram showing the correlation of protein expression levels in patients for whom both tissue and plasma samples were available.

To evaluate the ability of the assay to measure protein levels in plasma, we applied the assays to a panel of 45 plasma samples from patients with 3 different tumor types (breast, colon, ovarian) (**Figure 4A**). We used 100 µL aliquots of plasma as input for the assay. Each specimen was independently digested, enriched for the IO-1 analytes, and analyzed by LC-MRM. Specificity of endogenous peptide detection was assured by equal retention times and relative areas of light and heavy transitions. Overall, 38/52 peptides corresponding to 37 proteins were detected above LOQ in the plasma samples. The histogram of number of peptides detected across the panel showed that over half (n=24) of peptides were detected in all samples analyzed, with a small number of peptides detected below the LOQ in a subset of samples (**Figure 4B**) and 14 peptides not detected in any sample, likely due to the analytes not being secreted or shed to quantifiable levels. Compared to the variation seen in the tissue panel, there were less inter-individual differences in plasma, with the median inter-individual CV being 47% (range 10-152%). Like the tissue samples, unsupervised clustering of the expression levels for the peptides in the IO-1 multiplex panel (**Figure 4C**) did not separate the samples by tumor type.

Finally, there were 25 plasma samples for which we also analyzed tissue specimens from the same patient. The distribution of correlation coefficients for expression levels found in tissue and plasma from the same patient are plotted in **Figure 4D**. Overall, 31 peptides were detected in both tissue and plasma specimens, but there was poor intra-patient correlation between the two sample types. Based on previous results (108), we expected there may be high overlap in the peptides detected; however, it was unknown how the relative quantities may be correlated. Because protein abundance may also vary depending on specimen collection conditions (e.g., blood contamination in tissue specimens and/or cell shearing during plasma collection), we also examined if blood contamination in tissue specimens affected the correlation of peptides detected in tissue and plasma. A “blood contamination” score was assigned to each tissue specimen using the intensity of gel electrophoresis bands corresponding to the molecular weights of albumin and hemoglobin in each tissue lysate; however, correlation between tissue and plasma levels did not improve using only the samples with high “blood contamination” scores (data not shown).

### 3.5 The Antibodies Are a Useful Resource for Additional Applications

In parallel to their development for immuno-MRM, the new antibodies generated for this study were characterized for reactivity to the target proteins in traditional immunoassay approaches. Antibodies were tested for utility in traditional Western blotting or Wes system using purified recombinant proteins (where available) and/or lysates from cancer cell lines. Blots were deemed positive if a prominent band at the expected molecular weight (given by the vendor for the recombinant proteins or based on the molecular weight reported in UniProt.org) was observed. For this set of antibodies, 47% (14/30) of the antibodies tested (14/30) were positive against recombinant proteins, and 60% (12/20) were positive against lysates (not all antibodies that were negative for the recombinant protein were tested against cell line lysates). These results were consistent with expectations based on previous work (109). Antibodies scoring positive in Western blotting were tested for application in protein array using the NCI-60 cell line array. Overall, 3 of the antibodies tested were positive, based on normalized levels above background. The data are summarized in **Supplementary Table 7**. The monoclonal antibodies and characterization data are available as a resource for the research community *via* the CPTAC antibody portal (antibodies.cancer.gov; see **Supplementary Table 2**).

## 4 Discussion

We present the development and characterization of a multiplex panel of 52 assays for quantifying 46 immuno-modulatory proteins in tissue and plasma biospecimens. The panel shows excellent quantitative characteristics in tissue and plasma, with wide dynamic range and high precision. We used a panel of 180 biospecimens (135 tissue samples, 45 plasma samples) to demonstrate feasibility of endogenous measurements using the IO-1 immuno-MRM assay panel. In addition to high detection rates in frozen biopsies, the assays were successful in FFPE tissues, opening the possibility for retrospective studies.

The immuno-MRM approach offers sufficient sensitivity for analysis of low abundance proteins due to the ability of antibodies to enrich the target peptides from complex samples. Enrichment factors are analyte-dependent and range between 10^2^-10^4^ (110). The enriched sample contains a much-simplified background matrix of peptides, which improves ion suppression in the mass spectrometer. While the recovery efficiencies of the peptides in the enrichment step vary, the enrichment factors greatly overcome any sample losses associated with the additional sample handling (i.e., compared to direct analysis without enrichment), making the immuno-MRM approach highly advantageous for analysis of low abundance proteins and peptides.

Quantification of protein expression offers a powerful complementarity to existing technologies. Because mRNA does not always correlate, and thus fails to predict protein levels, the direct measurement of proteins gives a more accurate link to phenotypes. Likewise, the quantitative nature of the immuno-MRM panel offers a powerful complementarity to measurements performed by IHC. IHC is semiquantitative in nature, and quantification is susceptible to technical factors as well as subjective interpretation. However, IHC does provide useful spatial context for protein expression. The immuno-MRM measurements add complementary quantitative information that furthers the potential for useful interpretation of results by pathologists.

A large advantage of LC-MRM-MS is that analytes can be readily multiplexed. We used a panel of 50 monoclonal antibodies for enriching peptides from the digested samples. The high specificity of the mass spectrometer allows for analysis of much larger multiplex groups (compared to traditional immuno-assays), since interferences can be readily detected and usually avoided, and since MS-based assays have larger dynamic ranges compared with conventional immunoassays. Indeed, immuno-MRM assays have been demonstrated for panels up to 150 antibodies (28).

To produce actionable information from tumor biopsies, the assay must be sensitive enough to quantify protein networks in core needle biopsies or 5-10 micron sections cut from FFPE blocks. Although we have demonstrated that immuno-MRM produces sufficient sensitivity and specificity in these biospecimen types, the quality of specimens collected in clinical trials and across different sites are likely to produce many specimens with much more limited material. To address this problem, future work will focus on optimizing the sensitivity of the approach by improving analyte recovery through addition-only sample processing protocols and minimizing losses due to surface adsorption in reaction vessels and lab plastics. These advances will enable a greater diversity of specimens for analysis. Another challenge in making bulk tissue measurements is the need to understand the contribution of tumor heterogeneity to the measurements. Other bulk tissue assays have addressed tissue heterogeneity by requiring a minimum percent tumor cellularity (111). The tumors in this study were associated with a minimum cellularity requirement of ≥60% (by number). We have previously demonstrated that when cellularity is low, normalization proteins can be valuable in controlling for heterogeneity. For example, in measuring the HER2 protein in samples with low tumor cellularity, we were able to improve the concordance between an immuno-MRM assay and the predicate clinical IHC assay by normalizing the MRM measurements to GAPDH (31). Therefore, based on the HER2 study, we have included GAPDH in the final validated assay to accommodate future applications where tumor cellularity may be low.

These assays are made available to the research community as part of a larger effort under the National Cancer Institute’s Beau Biden National Cancer Moonshot [APOLLO network (44)] to quantify immuno-modulatory proteins using MRM-based assays. The monoclonal antibodies, characterization data, and SOPs are freely accessible to the research community through NCI’s CPTAC Assay Portal (assays.cancer.gov) (46, 47) and CPTAC Antibody Portal (antibodies.cancer.gov). Applied to clinical biospecimens, the assays have the potential for expanding correlative studies, establishing metrics of on-target inflammation and tumor response, or studying cancer immunity mechanisms to identify new therapeutic targets.

## Data Availability Statement

The names of the repository/repositories and accession number(s) can be found below: ProteomeXchange: accession number PXD029167 (doi: 10.6069/4wt1-t595) or follow the link: https://panoramaweb.org/IO1immunoMRM.url. 

## Ethics Statement

The studies involving human participants were reviewed and approved by Fred Hutchinson Cancer Research Center Institutional Review Office. The patients/participants provided their written informed consent to participate in this study.

## Author Contributions

AP conceived or designed the work. JW, RL, LZ, RS, AB, DH, UV, TW, JJK, RI, CL, OM, TL, SC, MT, TC, SH, SW, RR, JGK, JR, JAK, and CR acquired, analyzed, or interpreted the data. KM, ND, MB, HY, SA, EB, SG-B, WB, TH, HR, JW, and AP supervised studies. JW and AP drafted or substantively revised the work. All authors read and approved the final manuscript.

## Funding

This research has been funded in whole or in part with Federal funds from the National Cancer Institute (NCI), National Institutes of Health, under the NCI Beau Biden National Cancer Moonshot (Task Order No. HHSN26100025, Applied Proteogenomics Organizational Learning and Outcomes, under Contract No. HHSN261201500003I), the NCI Clinical Proteomics Tumor Analysis Consortium (CPTAC, grant no. U01CA214114), the NCI Academic Industrial Partnership (grant no. R01CA235575), the NCI Research Specialist program (grant no. R50CA211499), in part by the NCI CPTAC Antibody Characterization Program, and a generous donation from the Aven Foundation. This work was partially supported by the VA Office of Research and Development, Cooperative Studies Program (CSP). The views expressed are those of the authors and do not necessarily reflect the position or policy of the Department of Veterans Affairs or the United States government.

## Author Disclaimer

The content of this publication does not necessarily reflect the views or policies of the Department of Health and Human Services, nor does mention of trade names, commercial products, or organizations imply endorsement by the U.S. Government.

## Conflict of Interest

The authors declare that the research was conducted in the absence of any commercial or financial relationships that could be construed as a potential conflict of interest.

## Publisher’s Note

All claims expressed in this article are solely those of the authors and do not necessarily represent those of their affiliated organizations, or those of the publisher, the editors and the reviewers. Any product that may be evaluated in this article, or claim that may be made by its manufacturer, is not guaranteed or endorsed by the publisher.
