# Supplementary material for: Targeted Mass Spectrometry Enables Multiplexed Quantification of Immunomodulatory Proteins in Clinical Biospecimens
Source: Front Immunol. 2021 Nov 11;12:765898. doi: 10.3389/fimmu.2021.765898 (PMC8632241; doi:10.3389/fimmu.2021.765898)
Supplement: Supplementary file 1 [file DataSheet_1.docx]

Supplementary Material

# Supplementary Figures


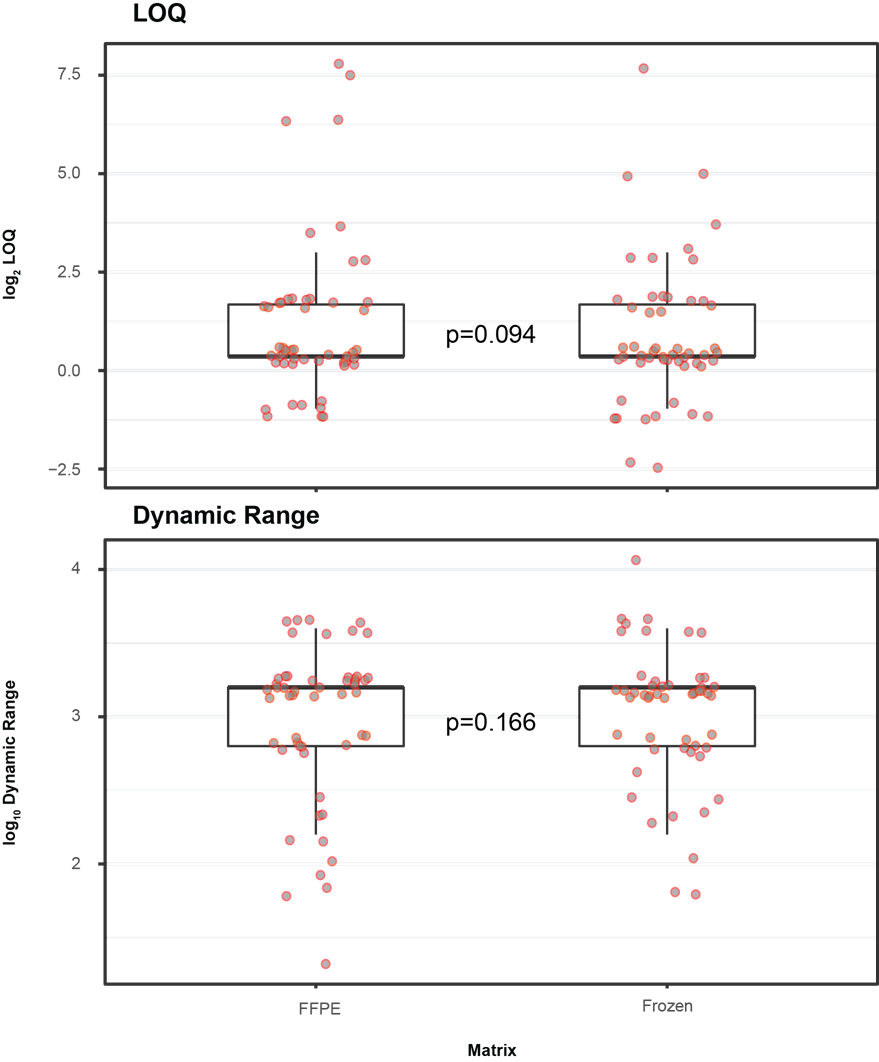


**Supplementary Figure 1.** Distribution of figures of merit (LOQ and dynamic range) for assays characterized in frozen and FFPE tissue matrices. Box plots show median (horizontal line), inner quartiles (box), 5-95% range (vertical lines). P-value shows the result of a two-sided t-test.


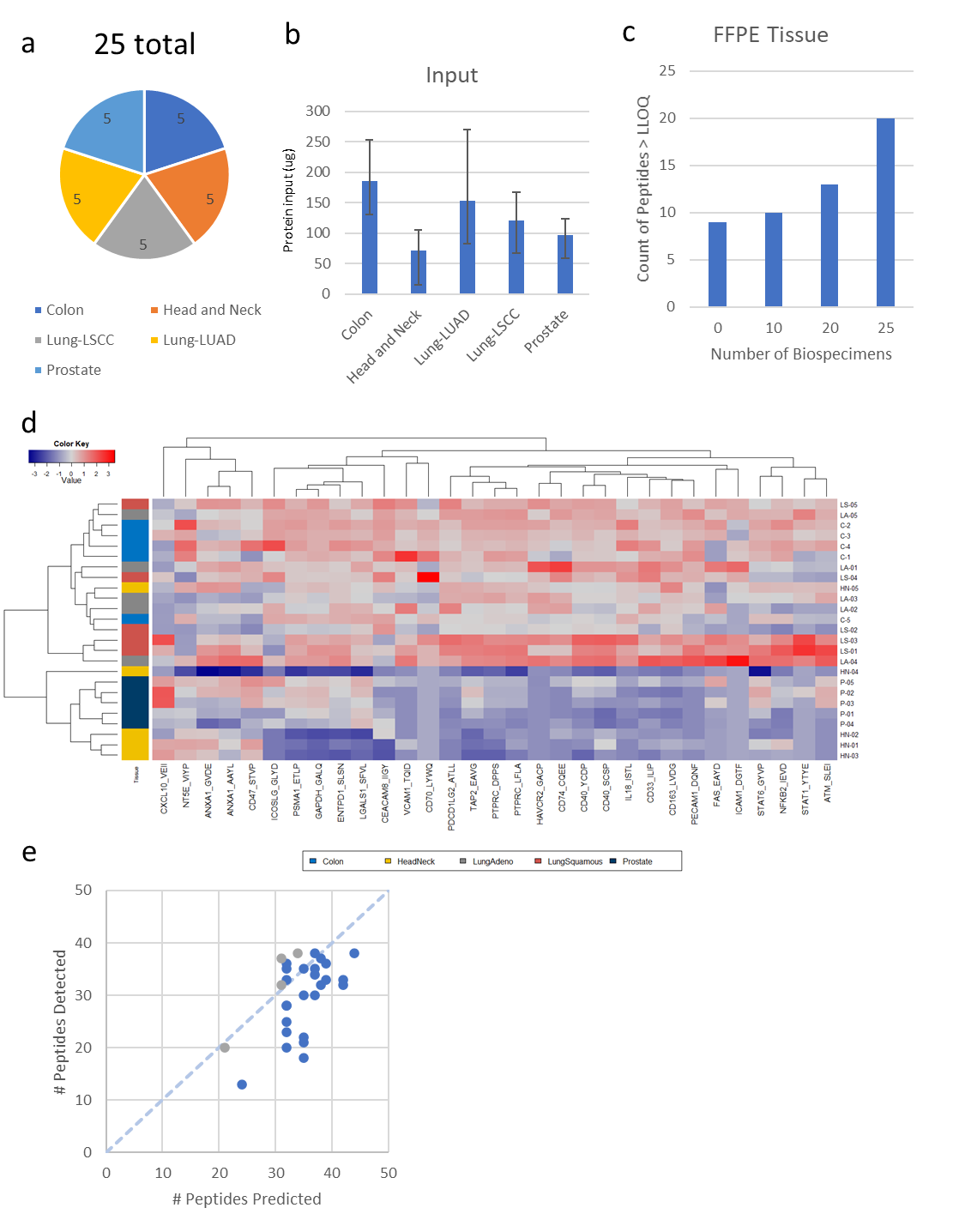


**Supplementary Figure 2.** (**a**) FFPE tissues were obtained for 25 tumors representing 5 tumor types, as indicated in the pie chart. (**b**) The amount of protein lysate extracted from 9 x10 μm sections. Error bars show the range in protein for the 5 specimens from each type. (**c**) Distribution of peptide detection plotted as a histogram, showing the number of peptides detected above LOQ across the 25 FFPE tumors. (**d**) Heatmap showing unsupervised clustering of analytes detected above LOQ in >50% of tumor specimens. Peak area ratios (light:heavy) were normalized for each peptide analyte, and the z-score was used for clustering. Peptide analyte labels represent gene symbol, followed by modification site (if applicable) and the first four letters of the peptide sequence. (**e**) Sample requirement predictions are accurate. Correlation of number of peptides detected in FFPE specimens (blue) and cell line lysates (gray) with the number of peptides predicted (based on minimal sample requirements determined from Figure 3). Predicted values for prostate specimens and cell line lysates were based on the average of all frozen tissues.
